# A new phase from compression of carbon nanotubes with anisotropic Dirac fermions

**DOI:** 10.1038/srep10713

**Published:** 2015-06-01

**Authors:** Xiao Dong, Meng Hu, Julong He, Yongjun Tian, Hui-Tian Wang

**Affiliations:** 1 MOE Key Laboratory of Weak Light Nonlinear Photonics and School of Physics, Nankai University, Tianjin 300071, China; 2 State Key Laboratory of Metastable Materials Science and Technology, Yanshan University, Qinhuangdao 066004, China; 3 Collaborative Innovation Center of Advanced Microstructures, Nanjing University, Nanjing 210093, China

## Abstract

Searching for novel functional carbon materials is an enduring topic of scientific investigations, due to its diversity of bonds, including *sp*-, *sp*^2^-, and *sp*^3^-hybridized bonds. Here we predict a new carbon allotrope, bct-C12 with the body-centered tetragonal *I*4/*mcm* symmetry, from the compression of carbon nanotubes. In particular, this structure behaviors as the Dirac fermions in the *k*_*z*_ direction and the classic fermions in the *k*_*x*_ and *k*_*y*_ directions. This anisotropy originates from the interaction among zigzag chains, which is inherited from (*n*, *n*)-naotubes.

Carbon has a large number of allotropes, including graphite, graphene, diamond, fullerenes, and carbon nanotubes (CNTs) under the ambient pressure, because it is able to form *sp*-, *sp*^2^-, and *sp*^3^-hybridized bonds. New functional carbon allotropes have been the focus of numerous theoretical and experimental explorations, because of not only their importance in basic science but also broad application prospects in technology.

In the past decades, the high pressure behaviors of carbon have a ttracted the attention of scientists. Compressing different carbon allotropes, such as graphite, fullerenes, and CNTs, can product various phases, such as diamond, superhard post-graphite phases^1^,^2^,^3^,^4^,^5^,^6^, unprecedented hard nanotwinned diamond^7^, fullerene polymers^8^, amorphous carbon^9^, and some elusive allotropes^10^,^11^,^12^. Meanwhile, some theoretical tools for predicting the structures and the kinetic process are necessary and have yielded great success, such as genetic algorithm^13^, basin hopping^14^,^15^, evolutionary metadynamics^16^, variable-cell nudged elastic band method^17^,^18^, and transition path sampling method^19^.

CNTs are a kind of specific one-dimensional carbon materials with outstanding mechanical and electronic characters. Since the band structures of CNTs can be considered as band-folding of graphene^20^, armchair (*n*, *n*) tube is metallic with one-dimensional Dirac fermions as the projection of graphene band structure. The high pressure behaviors of the CNT systems have attracted much experimental^21^ and theoretical^22^ interest, due to the complex and interesting phase transitions under high pressure. Pressure makes the tubes close to each other, and then the nearest neighbor carbon atoms among the adjacent tubes transit from *sp*^2^ to *sp*^3^ states and bond inter-cube. These novel metastable phases can be called as the single-walled CNT (SWCNT) polymers^22^. However, the polymer models did not consider the fact that the carbon atoms with *sp*^2^ and *sp*^3^ hybridization favors to possess different configurations, such as planar trigons and tetrahedrons. This will force to reconstruct the structures and then to happen more complex phase transition.

## Results

### Structure

**Figure 1 f1:**
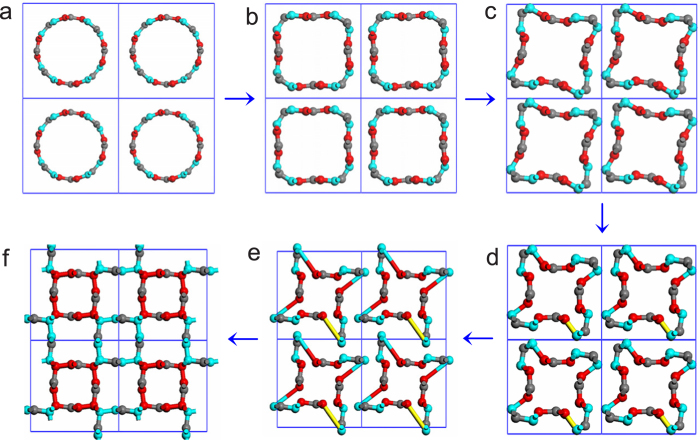
Formation process of bct-C12 from compression of (6, 6)-CNT at 40 GPa. Cyan atoms indicate the atoms connecting independent tubes in the compression procedure. Red atoms indicate the atoms which have new bonding in the procedure and yellow bonds show the broken bonds in **d** and **e**.

We use tetragonal stacking (6, 6)-CNTs as the precursors to perform the relaxation under different pressures and then to obtain the new carbon allotropes. When the pressure is changed from 40 to 60 GPa, the transition will fall into a trap of new phase, as the procedure shown in Fig. 1. Initially, the tubes approach to each other and there appear new connecting among the tubes as former paper^22^ presented (Fig. 1a,[Fig f1]c). Firstly the compression makes the original cylinder tubes (Fig. 1a) to become into the rounded squares (Fig. 1b). Then the newly formed *sp*^3^ carbon atoms (cyan) force the neighboring atoms (red) away from each other and the four edges of the rounded square become concaved (Fig. 1c). Meanwhile, the *sp*^2^ atoms favor to stay in a plane, which further exacerbates the concavity of the tubes (Fig. 1d). And then the tubes transform to pinwheel shapes (Fig. 1e). Finally, some bonds are broken and some new bonds are rebuilt, resulting in the formation of a new phase (Fig. 1f). Such a new structure can be considered as the second step product of the CNT compression with *sp*^2^ and *sp*^3^ hybridization reconfiguring the geometry, while the SWCNT polymers can be the first order product. Surprisingly, we also decompress to the atmospheric pressure, and find that it has low energy, good mechanic characters, and fantastic band structure, as the following discussion.

**Figure 2:**
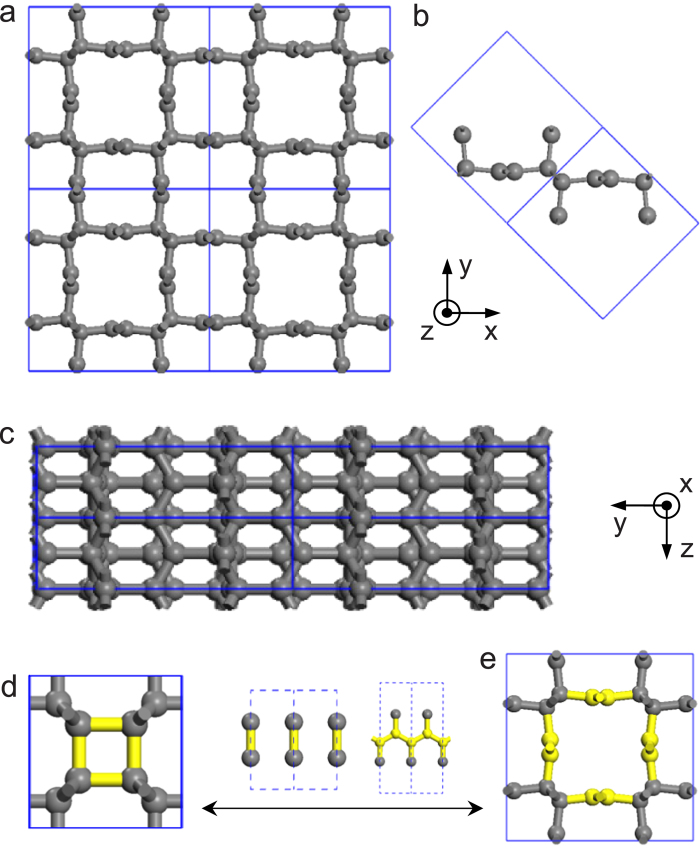
Geometric structure of bct-C12. **a, c** the conventional cell of bct-C12 from (0,0,1) direction and (1, 0, 0) direction. **b** the primitive cell of bct-C12. **d, e** the relationship between the structures of bct-C4 and bct-C12.

This new phase is identified to exhibit the body-centered tetragonal I4/*mcm* symmetry and is designated as the bct-C12, in which its conventional cell contains 24 atoms (Fig. 2) and its primitive cell has 12 atoms (Fig. 2b). At zero pressure, it has lattice parameters of *a* = *b* = 8.5 Å *a*nd *c* = 2.45 Å. Carbon atoms occupy the Wyckoff positions 8*h* (0.21, 0.29, 0) and 16*k* (0.73, 0.96, 0), respectively. In particular, the atoms located at the 8*h* Wyckoff positions have *sp*^3^ hybridization (painted in grey in Fig. 2e) and those occupying the 16*k* have *sp*^2^ hybridization (painted in yellow in Fig. 2e). Due to the same D_4h_ point group, the bct-C12 has much structural similarity to bct-C4 (refs. 5,6). It can be found that if we use zigzag carbon chains to replace the 4-ring of the bct-C4, the bct-C4 (Fig. 2d) becomes into the bct-C12 (Fig. 2d) in geometric configuration. However, the atoms in the additional zigzag chain are *sp*^2^ hybridization, while the atoms in the original bct-C4 are *sp*^3^ hybridization.

### Stability

**Figure 3:**
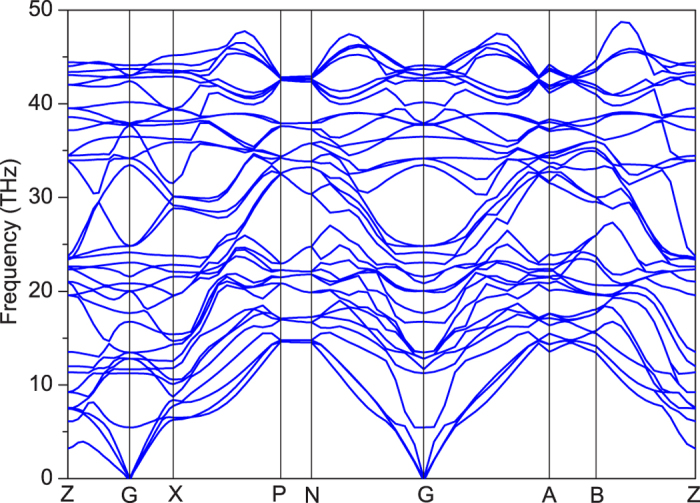
Phonon spectrum of bct-C12 at zero pressure.

The bct-C12, as a metastable phase of carbon at atmospheric pressure, has a formation energy of 0.18 eV/atom relative to graphite, while the bct-C4 is 0.26 eV/atom, the M-carbon 0.17 eV/atom, the (6,6)-CNT 0.13 eV/atom, and the fullerene C_60_ 0.39 eV/atom, respectively. One should be emphasized that the above results we calculated are similar to the previous calculations^3^,^5^,^6^,^23^. This means that the bct-C12 has the similar stability to the precursor (6, 6)-CNT and the superhard post-graphite phases, M-carbon, and the lower formation energy than bct-C4 and C_60_. In addition, the calculated phonon spectrum clearly indicates its dynamical stability (Fig. 3). Clearly, this phase is a novel material, even though the pressure is decompressed to the atmospheric pressure.

### Mechanical property

As shown in the calculation results, the bct-C12 has its bulk modulus of 315.9 GPa and shear modulus of 225.4 GPa. Based on the modified microscope model^24^,^25^,^26^, the calculated theoretical Vickers hardness is 31.6 GPa for the bct-C12. Although this hardness is much lower than 93.6 GPa of diamond, the bct-C12 is still a hard material with its Vickers hardness being slightly higher than 30.2 GPa of α-SiO_2_. In particular, the metallic property will dramatically reduce the hardness of material^26^, hence this hardness of the metallic bct-C12 should be quite high among the metallic materials. In addition, the bct-C12 has much interspace and its *sp*^3^ parts are not in a perfect tetrahedron. As a result, the structure is easy to slip. This is the reason why the bulk modulus of this structure is about 1.5 times of the shear modulus, which implies that this structure has better malleability than the superhard materials, such as diamond.

### Electronic property

**Figure 4:**
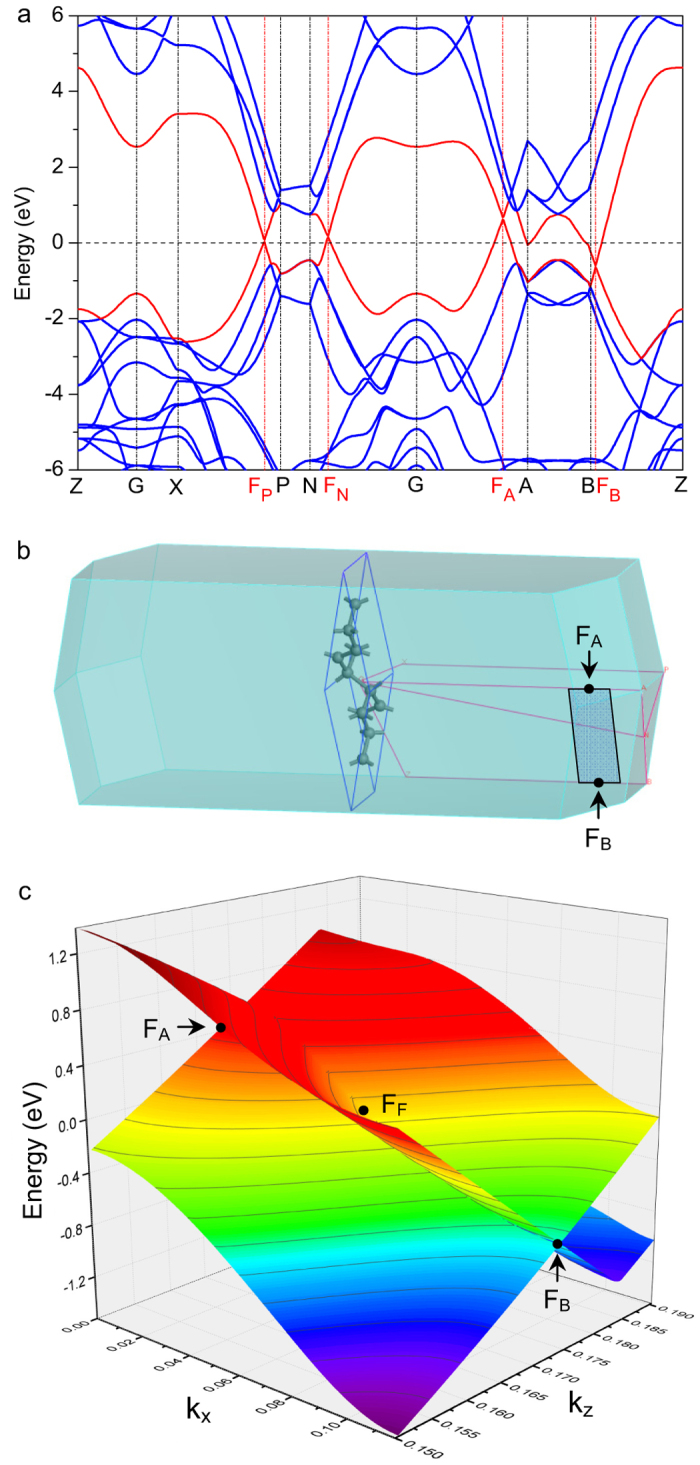
Electric structure of bct-C12. **a** the band structure of bct-C12. **b** the *k* path and Brillouin zone of bct-C12. **c**, the energy of the valence and conduction bands as a function of *k*_*x*_-*k*_*z*_ plane. The maximum and minimum in Dirac surface in energy, F_A_ and F_B_, are pointed out. F_F_ shows the intersection of Fermi surface and Dirac surface. Zero point of energy is Fermi level. The units of *k*_*x*_ and *k*_*z*_ are 2*π* Å^−1^.

The band structure (Fig. 4a) indicates the bct-C12 to be metallic. Since the *σ* bands of the *sp*^3^ atoms have the lower energy, the bands cross the Fermi level are the *π* bands around the *sp*^2^ zigzag chain (red lines in Fig. 4a). In particular, the band structure has a novel property that, along the *k*_*z*_ axis, the linear valence and conduction bands meet at a single point, which is similar to the Dirac point in graphene. These points of intersection depend on both *k*_*x*_ and *k*_*y*_, and show the energy fluctuation in Brillouin zone. The maximum and minimum of the points of intersection are 0.57 eV at F_A_ (0, 0, 0.172) × 2*π*Å^−1^ along the G^-^A line and −0.71 eV at F_B_ (0.117, 0, 0.174) × 2*π* Å^−1^ along the Z^-^B line, respectively. It should be noted that since F_A_ and F_B_ have nearly the same *z* coordinates, the surface composed of these Dirac points is close to a plane, which can be referred to as the Dirac surface.

We find a “weird” quasiparticle that exhibits a linear Dirac fermion behavior in the *k*_*z*_ direction, while a classic two-order dispersion in the *k*_*x*_-*k*_*y*_ plane, meaning that the quasiparticle can be changed from the Dirac fermions to the normal fermions by gradually changing the wavevector. As shown in Fig. 4c, in the *k*_*x*_-*k*_*z*_ plane, the band exhibits an anisotropic wedge instead of the cone in graphene.

In particular, at the F_A_ point, which is the top of this band, the slopes in the *k*_*z*_ direction is ±39.1 eV.Å, equivalent to a velocity 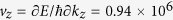 m/s, which is slightly bigger than that approaching the Dirac points of graphene, 34 eV.Å (*v* = 0.82 × 10^6^ m/s). At this points, we have *v*_*x*_ = *v*_*y*_ = 0. The second derivatives in the *k*_*x*_ and *k*_*y*_ directions are −775.2 eV.Å^2^ for the valence band and −407.2 eV.Å^2^ for the conductive band, which indicate the effective mass of electron 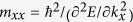 and 
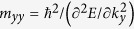
, correspondingly the effective mass is −0.39*m*_*e*_ for the valence band and −0.74*m*_*e*_ for the conductive band, where *m*_*e*_ is the true electron mass. By contrast, the effective mass in the *z* direction is *m*_*zz*_ = 0.

Meanwhile, the band bottom, F_B_ point, is also close to the boundary of Brillouin zone, which distorts its Dirac behavior in the *z* direction. The slopes are −51.4 and 37.1 eV∙Å, correspondingly the velocity *v*_*z*_ = 0.90 × 10^6^ m/s in the *+k*_*z*_ direction and −1.24 × 10^6^ m/s (more than 1.5 times of that in graphene) in the −*k*_*z*_ direction, while *v*_*x*_ = *v*_*y*_ = 0. In the *k*_*x*_-*k*_*y*_ plane, the curvatures of the valance and conductive bands are 308.0 and 707.0 eV.Å^2^, respectively, and then the corresponding effective mass is *m*_*xx*_ = *m*_*yy*_ = 0.98*m*_*e*_ for the valence band and *m*_*xx*_ = *m*_*yy*_ = 0.43*m*_*e*_ for the conductive band, while *m*_*zz*_ = 0.

Since the Dirac surface crosses the Fermi surface (*E* = 0), which is of great importance to electronic transport, we also analyze the band property of their intersections, for instance in F_F_ (0.054, 0, 0.170) × 2*π* Å−^1^. In the *k*_*z*_ direction, the slopes are −59.4 and 30.7 eV∙Å, correspondingly the velocity *v*_*z*_ = 0.74 × 10^6^ m/s in the *+k*_*z*_ direction and −1.43 × 10^6^ m/s in the *–k*_*z*_ direction, while *m*_*xx*_ = 0. In the *k*_*x*_ direction, F_F_ is not an extreme point, so its slopes are nonzero. The slope of −15.4 eV∙Å corresponds to the velocity *v*_*x *_= 0.37 × 10^6^ m/s in the −*k*_*x*_ direction. The curvatures of the valance and conductive bands are −188.9 and 84.0 eV∙Å^2^ at F_F_, respectively, corresponding to the effective mass of *m*_*xx*_ = *m*_*yy*_ = −1.59*m*_*e*_ for the valence band and *m*_*xx*_ = *m*_*yy *_= 3.58*m*_*e*_ for the conductive band. Because the band at F_F_ is smoother than F_A_ and F_B_, the quasi-particles are heavier.

By analyzing F_A_, F_B_ and F_F_, we find the anisotropy of the massless Dirac behavior in the *k*_*z*_ direction and the classic behavior in the *k*_*x*_ and *k*_*y*_ directions, is the intrinsic property of the Dirac surface in the Brillouin zone of bct-C12. It distributes in the energy space from −0.71 to 0.51 eV and is quite close to the Fermi level.

## Discussions

The unexpected anisotropic Dirac fermion originates from the anisotropy of effective mass of the quasiparticle. True particles have spatial rotation invariance, so their masses are scalar constants. But three dimensional periodic system breaks the rotation invariance, so the effective mass of the quasiparticle in crystals is described by a 3 × 3 matrix tensor. In the general case, the three eigenvalues approach to each other and we can average them to give the averaged effective mass of the quasiparticle. By contrast, the bct-C12 is an extreme case. Especially, the bct-C12 inherits the zigzag carbon chain from its precursor (6, 6)-CNT, so it is massless Dirac fermion in the *z* direction. Unlike the normal CNT, however, the distance between the zigzag chains is only 3.02 Å which is close enough for the interaction between the 2*p* electrons around the *sp*^2^ C atoms beyond the tubes, and the chain makes a common tetragonal lattice to have the classical band structure. As a result, it shows a strong anisotropy between the Dirac and classical behaviors.

Interestingly, although the bct-C12 is metallic, it should have the transport behavior like the semiconductor. Particularly, the F_A_ point (0.57 eV) is slightly above the Fermi level, while the F_B_ point (−0.71 eV) lies slightly below the Fermi level. So the Fermi level cross the Dirac surface (Fig. 4c), and the bct-C12 possesses the spontaneous electrons at F_A_ and holes at F_B_ in its ground state. Due to different signs of effective mass in the *x*-*y* plane, F_A_ and F_B_ are different doping and will obviously contribute differently to the electronic properties.

In conclusion, by compressing the (6, 6)-CNT, we discover a new tetragonal carbon phase, which can be quenchable when decompressing to zero pressure. It is metallic and as hard as α-quartz. Most strikingly, this structure has the anisotropy of the Dirac behavior in the *k*_*z*_ direction and the classic behavior in the *k*_*x*_ and *k*_*y*_ directions, which originates from the interaction between the Dirac zigzag chains. To our knowledge, this should be the first reported a system that has anisotropy in both Dirac and classic fermions. This research provides a new member of the big family of carbon allotropes and novel insight to their transport behaviors. Of course, this new phase maybe have some unexpected electronic behaviors.

## Methods

Carbon nanotubes packed in periodic crystal lattices with a standard intertube spacing of 3.4 Å were constructed using the Materials Studio package^27^. Structural relaxations and property calculations were performed based on the density functional theory (DFT) as implemented in the CASTEP code^27^. The Vanderbilt ultrasoft pseudopotential was used and the electron-electron exchange interaction was described by the local density approximation (LDA) exchange-correlation functional of Ceperley and Alder, as parameterized by Perdew and Zunger (CA-PZ)^28^,^29^. The plane-wave cutoff energy with 800 eV, and a *k*-point spacing (2*π* × 0.03 Å^−1^) was used to generate Monkhorst-Pack *k*-points grids for Brillouin zone sampling^30^. Primitive cells were used to calculate the band structures and the bulk modulus, and shear modulus. Vickers hardness is calculated using the modified microscopic model^24^,^25^,^26^. All the results were also confirmed by the all-electron projector augmented wave (PAW) method^31^ as implemented in the VASP code^32^. Phonon calculations were performed using the PHONOPY code^33^.

## Additional Information

**How to cite this article**: Dong, X. *et al*. A new phase from compression of carbon nanotube with anisotropic Dirac fermions. *Sci. Rep.* **5**, 10713; doi: 10.1038/srep10713 (2015).
